# Chronic myeloid leukemia with sleep-related painful erections as a first symptom: a case report

**DOI:** 10.1186/s13256-023-04222-3

**Published:** 2023-11-30

**Authors:** Yao-dong Han, Hong-jie Chen

**Affiliations:** 1 Department of Hematology, The First People’s Hospital of Lanzhou, Lanzhou, 730050 China; 2 Department of Urology, The First People’s Hospital of Lanzhou, Lanzhou, 730050 China

**Keywords:** Sleep-related painful erections, Chronic myeloid leukemia

## Abstract

**Background:**

Sleep-related painful erections are characterized by deep penile pain that occurs during erections in the rapid eye movement stage of sleep.

**Case presentation:**

This case presents a 43-year-old Chinese Han patient with sleep-related painful erections. Turgid painful erections (4–5 episodes of tumescence) during the sleep hours caused pain. Further, blood testing revealed an abnormal increase in white blood cells (123 × 10^9^/L). The patient was diagnosed with chronic myeloid leukemia by bone marrow biopsy, BCR::ABL1 fusion gene testing, and *Philadelphia chromosome*. However, the sleep-related painful erections have dramatically decreased in frequency of erectile pain after chemotherapy for Chronic myeloid leukemia in our case.

**Conclusion:**

We considered that the occurrence of sleep-related painful erections was related to chronic myeloid leukemia and the case might be secondary sleep-related painful erections.

## Background

Sleep-related painful erections (SRPE) is an uncommon parasomnia making up of nocturnal penile tumescence accompanied by pain that awakens the individual. SRPE has normal non-painful erections and no penile anatomic abnormalities are present when awake [1].

## Case presentation

**Fig. 1:**
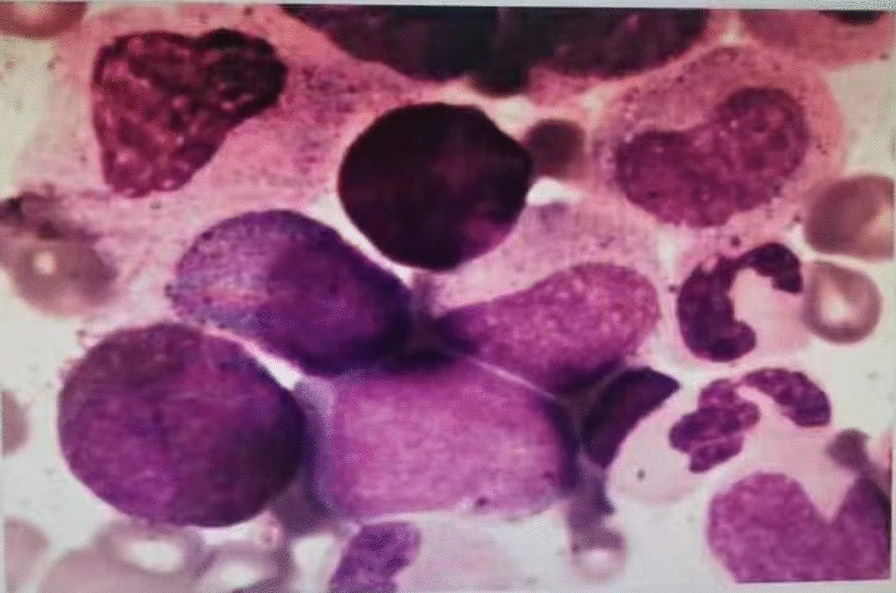
Percentage of granulocyte red showing as increased to 6.5:1; most of the proliferated granulocytes were intermediate granulocytes, late granulocytes, and rod-shaped nuclear granulocytes

**Fig. 2:**
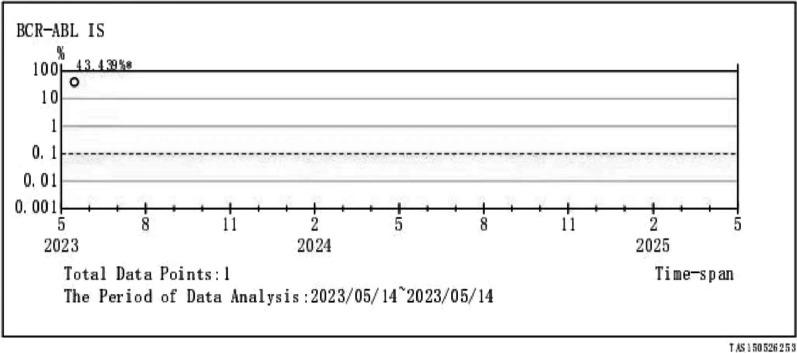
BCR-ABL fusion gene: P210 BCR/ABL (IS) showing as 43.439%

**Fig. 3:**
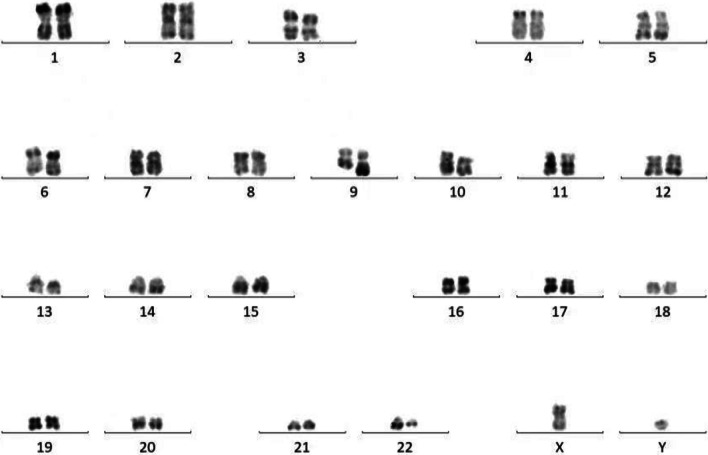
Ph chromosome: 46, XY, t (9; 22) (q34; q11) [10]; period of data analysis: 2023/05/14–2023/05/14

**Fig. 4:**
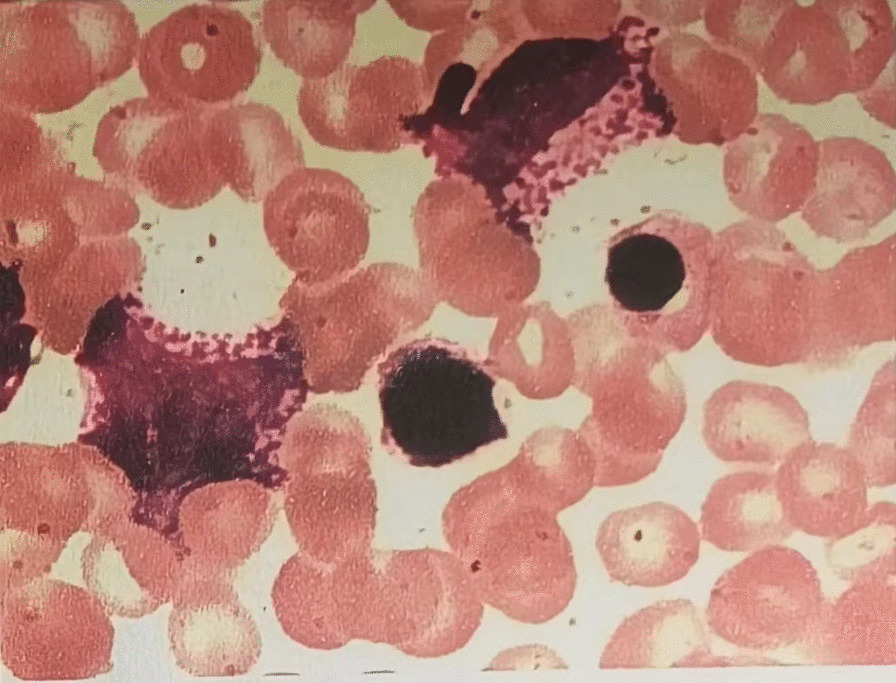
CML-complete response (CR )

**Fig. 5:**
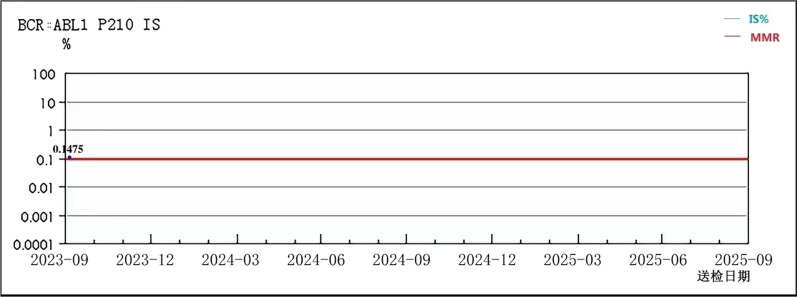
BCR-ABL fusion gene: P210 BCR/ABL (IS) showing as 0.1475%

**Fig. 6:**
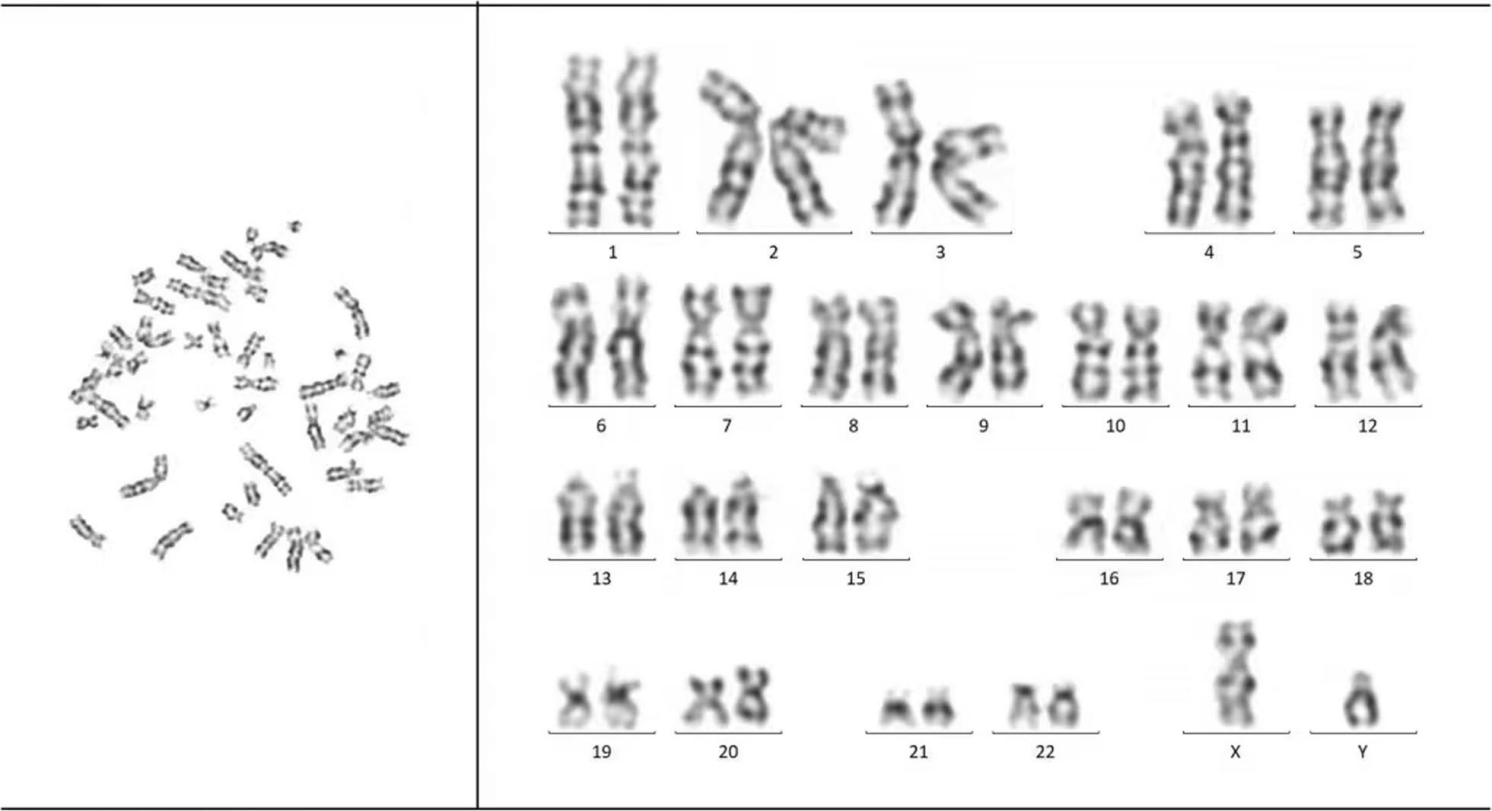
Ph chromosome: 46, XY [20]; period of data analysis: 2023/09/13–2023/09/13

A 43-year-old male Chinese Han patient reported a 9-month history of repeated nocturnal awakenings caused by painful erections after midnight, with an average of 4–5 episodes per week, lasting for 5–10 min. This pain that was located inside the erect penis could be relieved with urination, out-of-bed activity, and cooling the penis. He was able to fall back asleep only to have the pain wake him again. Sleep loss and poor-quality sleep caused excessive daytime sleepiness, anxiety, insufficient concentration, irritability, and extreme fatigue. The patient felt very miserable because it seriously affected his normal work and life. He reported being satisfied with his sexual life and had not noticed any negative change in the quality of his erections or ejaculations since the onset of symptoms. There was no context of a significant social, family, professional, or personal experience occurring before or at the onset of the symptoms. He had no history of hypertension, diabetes, urinary or sexually transmissible infections, and taking any testosterone supplements or sexual stimulants. Examination of the external genitalia was normal. Penile artery ultrasound and serum prostate-specific antigen and testosterone were normal. Physical and urinary examinations were performed, yielding results of no clinical relevance. Abdominal ultrasound showed a mild enlargement of the spleen (2 cm below rib margin). The full blood count result revealed an abnormal increase in white blood cells (White blood cell, WBC, 123 × 10^9^/L; Red blood cells, RBC, 5.21 × 10^12^/L; Platelets, PLT, 365 × 10^9^/L), and repeat check-up results were still unusual (WBC 125 × 10^9^/L, RBC 5.50 × 10^12^/L, PLT 359 × 10^9^/L). The patient was diagnosed with SRPE. Due to high suspicion of CML, he was then referred to the hematology department for a bone marrow cytology and Ph chromosome and BCR::ABL1 fusion gene testing. Bone marrow smear results were: percentage of granulocyte red increased to 6.5:1. Most of the proliferated granulocytes were intermediate granulocytes, late granulocytes, and rod-shaped nuclear granulocytes (Fig. 1). BCR::ABL1 fusion gene: P210 BCR/ABL (IS) was 43.439%, Ph chromosome: 46, XY, t (9; 22) (q34; q11) [10]. (Figs. 2, 3). The patient was diagnosed with chronic myeloid leukemia, chronic phase (Sokal score, low-risk group). The therapeutic goals of CML are to prolong survival, reduce disease progression, improve quality of life, and achieve treatment-free remission (that is, withdrawal). For this young patient who was expected to discontinue the drug, we chose the second-generation TKI drug (nilotinib 300 mg, twice a day), which can quickly obtain deep molecular response and reach the threshold of discontinuation. A total of 2 weeks after treatment, the patient’s leukemia count decreased (WBC 50 × 10^9^/L) and he was discharged. At 3-month follow-up, the frequency of erectile pain was significantly reduced from 4–5 to 0–1 times per week. In September, the patient’s leukocyte count decreased (WBC 4.6 × 10^9^/L) and bone marrow smear results were normal (Fig. 4). BCR::ABL1 fusion gene: P210 BCR/ABL (IS) was 0.1475% and Ph chromosome: 46, XY [20] (Figs. 5, 6). The patient is still being followed-up regularly.

## Discussion

In the present case, the patient saw a urologist for SRPE as first symptom. In fact, the actual prevalence of SRPE among patients seeking first-line urological advice was likely to be lower. We have not searched any reports of SRPE-associated leukemia from the literature. However, SRPE dramatically decreased in frequency after chemotherapy for CML in our case. We considered that the occurrence of SRPE was related to CML and the case might be secondary SRPE.

Sleep-related erections (SREs) naturally and involuntarily occur in all healthy men, usually during rapid eye movement (REM) sleep. Unfortunately, some men experience painful SREs that disrupt their sleep pattern: the so-called SRPE [2]. The International Classification of Sleep Disorders (ICSD) classifies SRPE by its severity as mild, moderate, and severe according to the frequency of occurrence, as described in Table 1 [3]. The prevalence of nocturnal painful erections is rare, occurring in fewer than 1/100 men presenting with sexual and erectile problems, with the overall mean age at onset for all reported cases being 40 years. Patients experience painful erections several times every night, each time lasting from minutes to an hour, starting from a long history of REM sleep deprivation to insomnia, disturbance of daytime performance, irritability, and reduced sex drive. Erections respond to non-medical attempts of detumescence using cold showers, walking, abduction and flexion of the hips, or urinating, which was described to be the most effective. Staying in bed usually makes the pain worse [4].

**Table 1:** Severity of sleep-related painful erections

Severity	Frequency of occurrence
Mild	Once per week
Moderate	Several times per week
Severe	Every night or several times per night

Even now, the phenomenon of SRPE has not been well understood [5]. Some investigators presumed a possible relation between SRPE and certain comorbidities, but overall SRPE appeared to be neither related to comorbidities nor associated with other sexual problems [6]. Wang *et al*. [7] use the PubMed database to obtain SRPE literature in 2021. The search terms used include sleep, painful, penis, and erection. After rigorous screening, the search returned 21 references published between 1987 and 2021. They summarized the pathogenesis of SRPEs and proposed the pathogenesis concept of “O-PAINT” where “O” represents obstructive sleep apnea (OSA) Syndrome, “P” represents psychological and spiritual factors, “A” is for androgen elevation, “I” is for compartment syndrome caused by ischemia, “N” is for neuroendocrine regulation, and “T” is for threshold of pain in the REM phase.

The common causes of secondary SRPE are hematopathy (chronic granulocyte, sickle cell, anemia), spinal cord injury or lesion, and other diseases with high blood viscosity [8]. Secondary SRPE is rarely reported because it is difficult to determine whether SRPE is secondary or coexisting. Szcs *et al*. [9] reported a case of SRPE associated with the posterior cerebral artery clearly compressing the inferior basal forebrain. They suggested that compression of the posterior cerebral artery stimulates and/or damages the inferior basal forebrain, leading to SRPE. It is likely vascular compression of the trigeminal nerve leads to trigeminal neuralgia. In Ferre’s report [3], three instances were undergoing continuous positive airway pressure (CPAP) treatments, during which the SRPE immediately dissipated, only to resume its usual frequency once CPAP had ceased, in conjunction with obstructive sleep apnea syndrome (OSAS). Zhang *et al*. [10] combined regular use of CPAP, tamsulosin (0.2 mg per night), alprazolam (0.8 mg/day), and escitalopram (20 mg/day) for an initial period of 1 month, which resulted in substantial improvement. Immediate effects included dramatic reduction of OSAS and daytime drowsiness with CPAP treatments (Apnea hypopnea index, AHI, 4.0/hour). Episodic SRPE became less frequent and the nocturia resolved. It cannot be determined whether SRPE and OSAS are secondary or coexisting relationships, but treatment of OSAS can contribute to the improvement of SPRE symptoms. Barnhoorn *et al*. [11] report a case of SRPE that only, but always, occurred after sexual intercourse with ejaculation the evening before. They do not have an explanation as to why the phenomenon in this patient only occurred after having had intercourse the night before. It could be that the deflating mechanism in this patient is deficient. A nonpharmacological solution was found in shifting the time of sexual intercourse.

CML-related SRPE may be closely related to leukemia cell infiltration, pelvic splanchnic nerve (erectile nerve) damage, and leukocytosis. There are some similarities between patients with SRPE and those with so-called stuttering priapism (also known as intermittent or recurrent priapism). The European guideline states that the etiology of stuttering priapism is similar to that of ischemic priapism, with sickle cell disease and leukemia being the most common causes [12]. In the present case, symptoms of SRPE were moderate, and relieved when CML was improved during a 3-month follow-up. Therefore, no drug intervention was given for the symptoms of SRPE. Follow-up is continuing.

## Conclusion

Secondary SRPE is rare disorder that may lead to missed diagnosis of primary disease or delayed diagnosis of SRPE. Secondary SRPE is mainly used to treat primary disease. The diagnosis of SRPE should be confirmed again or combined therapy should be given if the SRPE symptoms are not relieved after primary disease improvement.

## Data Availability

Not applicable.

## Abbreviations

SRPE: Sleep-related painful erections
REM: Rapid eye movement
CML: Chronic myeloid leukemia
SREs: Sleep-related erections
ICSD: International Classification of Sleep Disorders
CPAP: Continuous positive airway pressure
OSAS: Obstructive sleep apnea syndrome
